# Disseminated histoplasmosis in an immunocompetent individual diagnosed with gastrointestinal endoscopy: a case report

**DOI:** 10.1186/s12879-019-4542-x

**Published:** 2019-11-21

**Authors:** Yini Dang, Longfeng Jiang, Jianfu Zhang, Beijing Pan, Guoqin Zhu, Feipeng Zhu, Zhe Guo, Biao Wang, Guoxin Zhang, Yali Weng, Jun Li

**Affiliations:** 1 Department of Infectious Diseases, The First Affiliated Hospital of Nanjing Medical University, No.300 of Guangzhou Road, Nanjing, 210029 China; 2 Department of Gastroenterology, The First Affiliated Hospital of Nanjing Medical University, Nanjing, China; 3 Department of Hematology, The First Affiliated Hospital of Nanjing Medical University, Nanjing, China; 4 Department of Pathology, The First Affiliated Hospital of Nanjing Medical University, Nanjing, China; 5 Department of Geriatric Gastroenterology, The First Affiliated Hospital of Nanjing Medical University, Nanjing, China; 6 Department of Medical Imaging, The First Affiliated Hospital of Nanjing Medical University, Nanjing, China; 7 Department of nuclear medicine, The First Affiliated Hospital of Nanjing Medical University, Nanjing, China; 8 Department of Pharmacy, The First Affiliated Hospital of Nanjing Medical University, Nanjing, China

**Keywords:** Disseminated histoplasmosis, Endoscopy, Esophagus, Colon

## Abstract

**Background:**

Histoplasmosis is one of the invasive fungal infections and presents with symptoms mainly in the lungs. Disseminated histoplasmosis (DH) is rare and its lesions in the gastrointestinal tract are even uncommon. The concomitant involvement of the upper and lower gastrointestinal tract has never been described in the immunocompetent individuals.

**Case presentation:**

A 44-year-old immunocompetent Chinese man presented with fever, hepatosplenomegaly, fungal esophagitis and protuberant lesions with central depression and erosion along the mucous membrane of the colon. The patient was diagnosed as disseminated histoplasmosis by gastrointestinal endoscopy.

**Conclusions:**

Histoplasmosis should be taken caution in patients with fever and hepatosplenomegaly. Actions should be taken to avoid its disseminated infection associated high mortality.

## Background

Histoplasmosis is one of the invasive fungal infections and presents with symptoms mainly in the lungs [1]. Disseminated histoplasmosis (DH) is rare and its lesions in the gastrointestinal tract are even uncommon [1–3]. The concomitant involvement of the upper and lower gastrointestinal tract has never been described in the immunocompetent individuals. Here, a case of disseminated histoplasmosis in the non-endemic area was presented in an immunocompetent patient diagnosed by gastrointestinal endoscopy.

## Case presentation

A 44-year-old Chinese man admitted to Chaohu Hospital of Anhui Medical University in April 3rd 2018 and presented with intermittent high-grade fever (Tmax of 39.5 °C) with chills and rigor since January 2018. Before his admission to this hospital, he was treated with outpatient intermittent cephalosporin therapy, however no obvious curative effect was observed. After admission, ultrasonography and CT both revealed hepatosplenomegaly, and bone marrow tests demonstrated macrophages which abnormally engulf red blood cells and platelets and thrombocytopenia. Then this patient received piperacillin/tazobactam 3.375 g/q8h for 5 days and no improvement was observed. Afterwards the antibiotics were upgraded to biapenem 0.3 g/qd combined with teicoplanin 0.2 g/qd for 5 days. During his stay in Chaohu Hospital of Anhui Medical University, the patient lost 8 kg of weight and no significant curative effect was achieved. Due to this condition, the patient was transferred to the First Affiliated Hospital of Nanjing Medical University.

On his admission to our hospital, vital signs showed temperature of 39.4 °C, heart rate of 110/min, respiration of 22/min and blood pressure of 110/68 mmHg. The patient showed no palpable lymph nodes on the neck, the liver was 12 cm below the right rib border and 8 cm below the xiphoid, spleen was 11 cm below the left rib border. Medical history suggested there was no underlying disease, no smoking, no alcohol or illicit drug abuse.

Laboratory examinations on admission revealed hemoglobin of 112 g/L, red blood cell (RBC) counts 4.41 × 10^12/L, platelet counts 56 × 10^9/L and white blood cell (WBC) counts 3.62 × 10^9/L, liver enzymes γ-GGT 98.4 U/L, ALP 329 U/L, TBil 35.4 μmol/L, DBil 14.0 μmol/L, UBil 21.4 μmol/L, inflammatory markers (PCT 0.26 ng/mL, CRP 34.0 mg/L, FERR 1101.8 ng/mL), G test of 238.1 pg/mL, GM test of 0.375, immunoglobulin levels comprised IgG 11.7 g/L, IgA 1.23 g/L, IgM 2.9 g/L, normal complement levels with C3 0.71 g/L, C4 0.167 g/L. Lymphocyte subsets comprised 67% total T cell, 29% Helper T cell, 33% Suppressor T cell, CD4+/CD8+ 0.89, total T cell count 323 cells/μl, Helper T cell count 148 cells/μl, Suppressor T cell count 167 cells/μl, 3.7% early activated stage T cell, 6.91% middle activated stage T cell, 44.42% late activated stage T cell (see Additional file 1: Table S1).

No significant change was detected in terms of other blood tests including kidney function tests, tumor markers (AFP, CEA, CA199, CA-724, CYFRA21-1, NSE, TPSA, FPSA), autoantibodies, antibodies related to infectious diseases (HBV, HCV, HIV, CMV-DNA, EBV-DNA, T-SPOT).

**Fig. 1:**
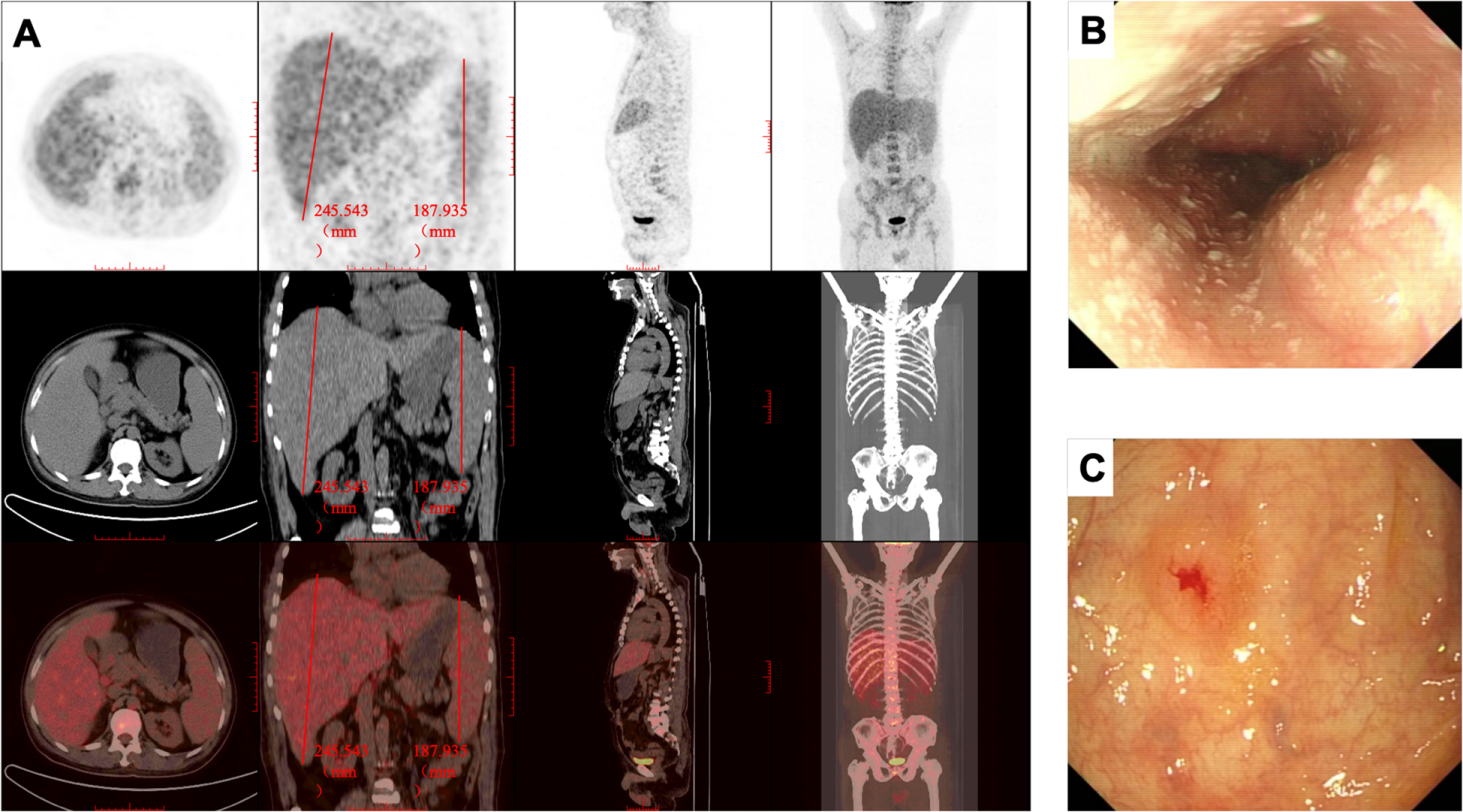
**a** Increased FDG uptake in liver and spleen; the length of liver was 245.543 mm and the length of spleen was 187.935 mm in the ^18^F-FDG PET/CT images before intervention. **b** esophageal mucosa was covered consecutive sheets of white bean curd-like substance under gastrointestinal endoscopy. **c** colon mucosa studded with protuberant lesions with central depression and erosion under gastrointestinal endoscopy

Ultrasonography revealed hepatosplenomegaly. The whole body was scanned with ^18^F-fluorodeoxyglucose (FDG) positron emission tomography with computed tomography (^18^F-FDG PET/CT) and showed that the length of liver was 245.543 mm and length of spleen was 187.935 mm. The enlarged liver and spleen showed mild increased metabolic activity according to the results of ^18^F-FDG PET/CT. Diffused slight hyper-metabolism in whole body bone marrow and multiple lymph nodes around porta hepatis and retroperitoneum were also depicted. As a 0.8 cm polyp-like lesion was detected in the small intestine with increased metabolism in the FDG PET/CT images, further gastrointestinal endoscopy was arranged (see Fig. 1a). PET/CT showed no significant central nervous system involvement and no lesion in lung, therefore a lumbar puncture and percutaneous lung puncture biopsy were not conducted.

**Fig. 2:**
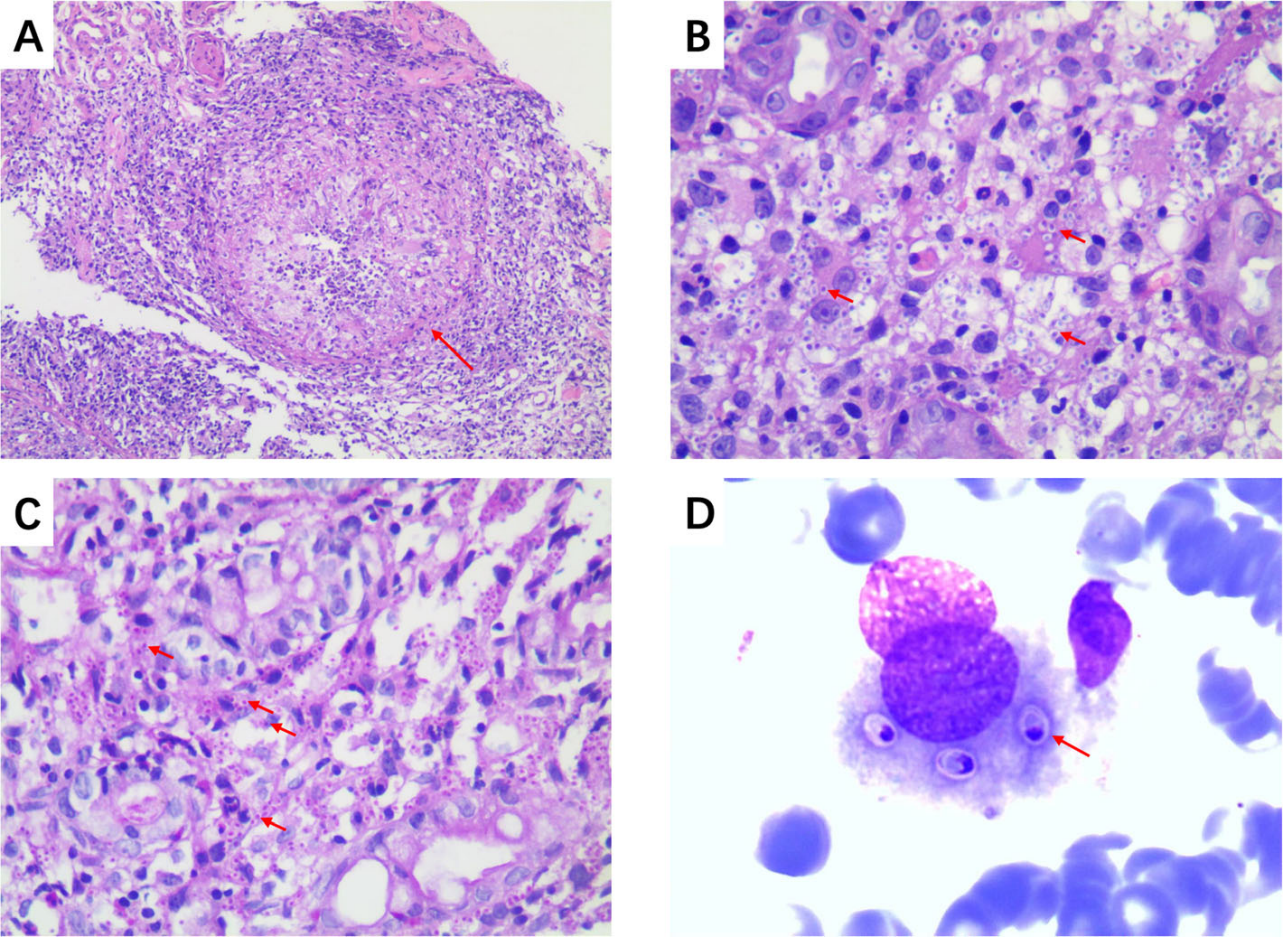
**a** Granuloma formation (red arrow, × 400). **b** HE stain of bone marrow puncture images before treatment (× 1000). **c** PAS stain (red arrows, × 400). **d** HE stain of bone marrow puncture images before treatment (× 1000)

Gastroscopy showed consecutive sheets of white bean curd-like substance attached to the esophageal mucosa, referring fungal esophagitis (see Fig. 1b). Colonoscopy showed protuberant lesions with central depression and erosion along the mucous membrane of the colon (Fig. 1c). Biopsy specimens of the colon identified numerous yeast-like structures containing increased numbers of histiocytes staining positive for PAS stain, indicating granulomatous inflammation induced by mycotic infection (see Fig. 2a, b and c).

Blood Routine, Biochemical Test, FERR were repeated during hospitalization and revealed progressive pancytopenia (see Additional file 1: Table S2). Hematoxylin and eosin stained bone marrow demonstrated oval or round organisms with amaranth nuclei and capsule-like unstained halos around these organisms observed in the cytoplasm of phagocytes. These observations were highly recommended of histoplasmosis capsulatum (see Fig. 2d).

The clinical manifestation, medical history, physical examinations and the related examinations confirmed his diagnosis of disseminated histoplasmosis involved in the digestive tract.

The patient started to receive intravenous amphotericin B deoxycholate at an initial dose of 7 mg/d in Day 1, 30 mg/d in Day 2, 50 mg/d in Day 3 and then every 5 mg/day was added till the dose reached 110 mg/d (approximately 1.5 mg/kg) [4]. In Day 25, the follow-up CT scan showed the size of liver and spleen narrowed (see Additional file 2: Figure S1). Gastrointestinal endoscopy showed esophageal and colon mucosa was normal as well (Additional file 2: Figure S2). Residual fungal cell walls were seen in both liver tissues and colon mucosa pathology specimens (see Additional file 2: Figures S3 and S4). H. capsulatum cannot be detected in the bone marrow aspirate (see Additional file 2: Figure S5). In Day 26, a total dose of amphotericin B deoxycholate reached 2400 mg, then it was replaced with itraconazole 200 mg bid orally. Two days after amphotericin B deoxycholate treatment, his temperature returned to normal. The liver narrowed to 7 cm below the right rib border and 4 cm below the xiphoid, the spleen was 6 cm below the left rib border in Day 36. The liver narrowed to 3 cm below the right rib border and the spleen was 3 cm below the left rib border in Day 44. In addition, the results of Blood Routine returned to normal (see Additional file 1: Table S1).

## Discussion and conclusion

According to the literature, only nine DH cases in the digestive tract of immunocompetent patients have been reported, among which none of the cases reported concomitant involvement of esophagus and colon (see Table 1) [2, 5–9]. This is a rare and typical DH case encroaching on esophagus, colon, liver, spleen and bone marrow in an immunocompetent individual. In this case, underlying chronic disease or underlying immunodeficiency was not observed. Results of laboratory examinations, bone marrow aspirate, PET/CT, gastrointestinal endoscopy and pathological examinations were collected before and after intervention. Significant improvement was observed with the application of amphotericin B deoxycholate. However, the transjugular liver puncture cannot be performed due to the limited technical support and the percutaneous liver puncture was not carried out due to his extremely low platelet level.

**Table 1:** Disseminated histoplasmosis involved in digestive tract in immunocompetent individuals

Articles	Country	Gender	Age	Symptoms	Involvement	Intervention	Prognosis
Yang 2013 [5]	China	Male	33	Fever, weight loss	Bone marrow, spleen, colon	Amphotericin B deoxycholate for a total of 2 g, shifted to oral itraconazole (200 mg bid)	Recovery
Badyal 2013 [6]	India	Male	62	Abdominal distension, abdominal pain, constipation	Colon	Tazocin 4.5 g and metronidazole 500 mg q8h, amphotericin B 500 mg and itraconazole 200 mg qd for 15 days	Recovery
Chaudhari 2013 [7]	USA	Female	22	Fever, weight loss, dysphagia,	Esophagus	Thoracotomy	Recovery
Wu 2015 [2]	China	Male	29	Fever, cough	Colon	Not reported	Not reported
Zhu 2016 [8]	China	Male	61	Abdominal pain, abdominal distention, anorexia	Colon	No anti-fungal drugs	Recovery
		Male	33	Fever, anorexia, pharyngalgia, cough, expectoration, weight loss	Bone marrow, colon, terminal ileum	Amphotericin B deoxycholate for a total of 1.47–2.79 g, shifted to itraconazole (200 mg bid) for 6 m	Recovery
		Male	59	Fever, abdominal pain, abdominal distention, anorexia, weight loss	Bone marrow, colon, terminal ileum	Amphotericin B deoxycholate for a total of 1.47–2.79 g, shifted to itraconazole (200 mg bid) for 6 m	Recovery
		Male	28	Fever, abdominal pain, abdominal distention, diarrhea, weight loss	Bone marrow, colon, terminal ileum	Amphotericin B deoxycholate for a total of 1.47–2.79 g, shifted to itraconazole (200 mg bid) for 6 m	Lost
Sharma 2017 [9]	India	Male	45	Right-sided pleural effusion	Lung, ileum	Amphotericin B deoxycholate, details not reported	Recovery

The diagnosis of this case was based on the observation of H. capsulatum in both colon mucosa pathology and bone marrow aspirate. Colon biopsy specimens showed granuloma formation. According to the previous study, only 8% of histoplasmosis showed fully developed granuloma, suggesting that this patient is an immunocompetent host. Furthermore, ^18^F-FDG PET/CT also suggested hematological disease in this case rather than infectious disease. ^18^F-FDG accumulated not only in malignant tumors but also in both infectious and non-infectious inflammatory lesions. There were overlaps between FDG uptake of malignant lesions and certain infectious processes due to the presence of macrophages. Tuberculosis and histoplasmosis infections lead to active granulomatous processes and may mimic malignant lesion with accumulated FDG uptake [10, 11]. Therefore, it is difficult to identify malignant tumors from infectious inflammatory lesions. It is the reason that this case was diagnosed with pathological examinations instead of ^18^F-FDG PET/CT. In addition, PCR and specific antigen test cannot be performed since there was limited technical support in our hospital. However, it is strongly suggested to perform PCR and specific antigen test in the future to further support the diagnosis of this disease [12].

This patient lived in a non-endemic area and visited Vietnam 8 years ago. Immunocompetent adults who exposed to the infectious organisms, the important risk factor for DH, may progress slower than immunocompromised or immunosuppressed individuals [13]. To the best of our knowledge, one of the studies reported that DH was diagnosed after 40 years [14]. Therefore, the natural history of this disease may be caused by the accumulation of fungal organisms in different organs or tissues, and it can be trigged by the variation of the immune micro-environment.

## Supplementary information

**Additional file 1.** Laboratory examination during the process of diagnose and intervention. Summary of laboratory examination during the process of diagnose and intervention.

**Additional file 2.** CT images, gastrointestinal endoscopy images, colonic biopsy images, liver biopsy images and bone marrow puncture images after intervention.

## Data Availability

All available data is presented within the manuscript and additional supporting files.

## Abbreviations

^18^F-FDG PET/CT: ^18^F-fluorodeoxyglucose positron emission tomography with computed tomography
AFP: Alpha-fetoprotein
CA199: Carbohydrate antigen 19–9
CA-724: Carbohydrate antigen 724
CEA: Carcinoembryonic antigen
CRP: C-reactive protein
CYFRA21-1: Cytokeratin 19 fragments
DBil: Direct bilirubin
DH: Disseminated histoplasmosis
FERR: Serum ferritin
FPSA: Free prostate specific antigen
HBV: Hepatitis B virus
HCV: Hepatitis C virus
HIV: Human immunodeficiency virus
NSE: Neuron-specific enolase
PAS stain: Periodic Acid-Schiff stain
PCT: Procalcitonin
RBC: Red blood cell
TBil: Total bilirubin
TPSA: Total prostate specific antigen
T-SPOT: Enzyme-linked immunospot assay
UBil: Undirect bilirubin
WBC: White blood cell
