# Supplementary material for: Disseminated histoplasmosis in an immunocompetent individual diagnosed with gastrointestinal endoscopy: a case report
Source: BMC Infect Dis. 2019 Nov 21;19:992. doi: 10.1186/s12879-019-4542-x (PMC6873732; doi:10.1186/s12879-019-4542-x)
Supplement: Supplementary file 1 — Additional file 1. Laboratory examination during the process of diagnose and intervention. Summary of laboratory examination during the process of diagnose and intervention. [file 12879_2019_4542_MOESM1_ESM.docx]

**Table S1. Laboratory examination on admission**

|  | Abnormal Values | Normal Rages |
| --- | --- | --- |
| Blood Routine | **Hemoglobin: 112g/L**  **RBC counts: 4.41×10^12/L**  **Platelet counts 56×10^9/L**  **WBC counts 3.62×10^9/L** | Hemoglobin: 130-175g/L  RBC counts: (4.30-5.80)×10^12/L  Platelet counts (125-350)×10^9/L  WBC counts (3.50-9.50)×10^9/L |
| Liver Function | **γ-GGT 98.4U/L**  **ALP 329U/L**  **TBil 35.4μmol/L**  **DBil 14.0μmol/L**  **UBil 21.4μmol/L** | γ-GGT 10.0-60.0U/L  ALP 30-120U/L  TBil 5.1-19.0μmol/L  DBil 0.0-6.8μmol/L  UBil 0.0-20.0μmol/L |
| Inflammatory Markers | **PCT 0.26ng/mL**  **CRP 34.0mg/L**  **FERR 1101.8ng/mL** | PCT 0.00-0.05ng/mL  CRP 0.0-8.0mg/L  FERR 23.9-336.2ng/mL |
| G test | **238.1pg/mL** | 0-100pg/mL |
| GM test | 0.375 | 0-0.5 |
| Immunoglobulin and Complement Levels | IgG 11.7g/L  IgA 1.23g/L  **IgM 2.9g/L**  **C3 0.71g/L**  C4 0.167g/L | IgG 1 7.0–16.0g/L  IgA 0.70–4.0g/L  IgM 0.4–2.3g/L  C3 0.9-1.80g/L  C4 0.10-0.4g/L |
| Lymphocyte subsets | Total T cell 67%  **Helper T cell 29%**  Suppressor T cell 33%  **CD4+/CD8+ 0.89**  **Total T cell count 323 cells/μl**  **Helper T cell count 148 cells/μl**  **Suppressor T cell count 167 cells/μl**  **Early activated stage T cell 3.7%**  Middle activated stage T cell 6.91%  **Late activated stage T cell 44.42%** | Total T cell 56-85%  Helper T cell 30-54%  Suppressor T cell 15-34%  CD4+/CD8+ 1.2-2.0  Total T cell count 955-2860cells/μl  Helper T cell count 556-1440cells/μl  Suppressor T cell count 320-1250cells/μl  Early activated stage T cell 0.5-3.5%  Middle activated stage T cell 5.1-10.1%  Late activated stage T cell 6.4-43.4% |

**Table S2. Laboratory examination during the process of diagnoses and treatment**

|  |  | Blood Routine | | | | Biochemical Test | | | | | FERR  (ng/mL) |
| --- | --- | --- | --- | --- | --- | --- | --- | --- | --- | --- | --- |
|  |  | WBC  (10^9/L) | RBC  (10^12/L) | Hb  (g/L) | PLT  (10^9/L) | ALT  (U/L) | AST  (U/L) | γ-GGT  (U/L) | ALP  (U/L) | ALB  (g/L) |  |
| Diagnoses | 04-17 | 3.62 | 4.41 | 112 | 56 | 30.5 | 23.5 | 98.4 | 329 | 33.8 | 1101.8 |
|  | 04-23 | 3.09 | 3.75 | 98 | 39 | 41.1 | 20.8 | 130.1 | 231 | 27.0 | 802.70 |
|  | 04-27 | 3.78 | 3.54 | 92 | 20 | 43.1 | 10.4 | 159.9 | 172 | 29.0 | 813.90 |
|  | 05-02 | 2.95 | 3.30 | 86 | 14 | 18.0 | 9.9 | 137.9 | 130 | 24.0 | 1035.80 |
| Treatment | 05-09 | 2.33 | 3.44 | 89 | 25 | 13.4 | 10.9 | 136.4 | 172 | 29.6 |  |
|  | 05-15 | 2.95 | 3.36 | 90 | 135 | 8.5 | 12.0 | 83.7 | 134 | 34.5 | 974.10 |
|  | 05-23 | 3.78 | 4.26 | 118 | 156 | 12.4 | 16.6 | 80.5 | 139 | 38.2 |  |
