# Supplementary material for: Disseminated histoplasmosis in an immunocompetent individual diagnosed with gastrointestinal endoscopy: a case report
Source: BMC Infect Dis. 2019 Nov 21;19:992. doi: 10.1186/s12879-019-4542-x (PMC6873732; doi:10.1186/s12879-019-4542-x)
Supplement: Supplementary file 2 — Additional file 2. CT images, gastrointestinal endoscopy images, colonic biopsy images, liver biopsy images and bone marrow puncture images after intervention. [file 12879_2019_4542_MOESM2_ESM.pdf]

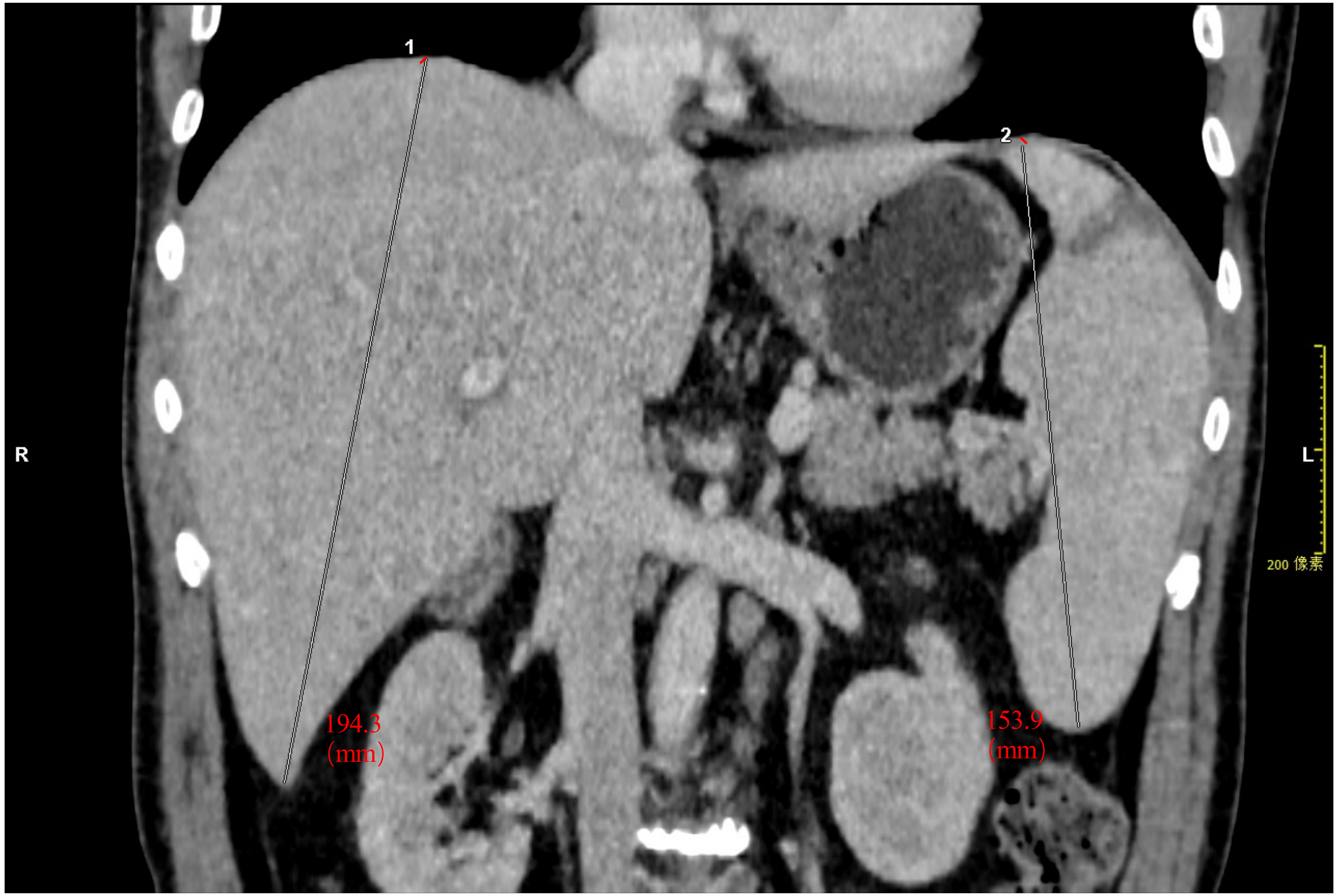

Figure S1. Figure S1. CT images after treatment. The length of liver was 194.3mm and The length of spleen was 153.9mm.

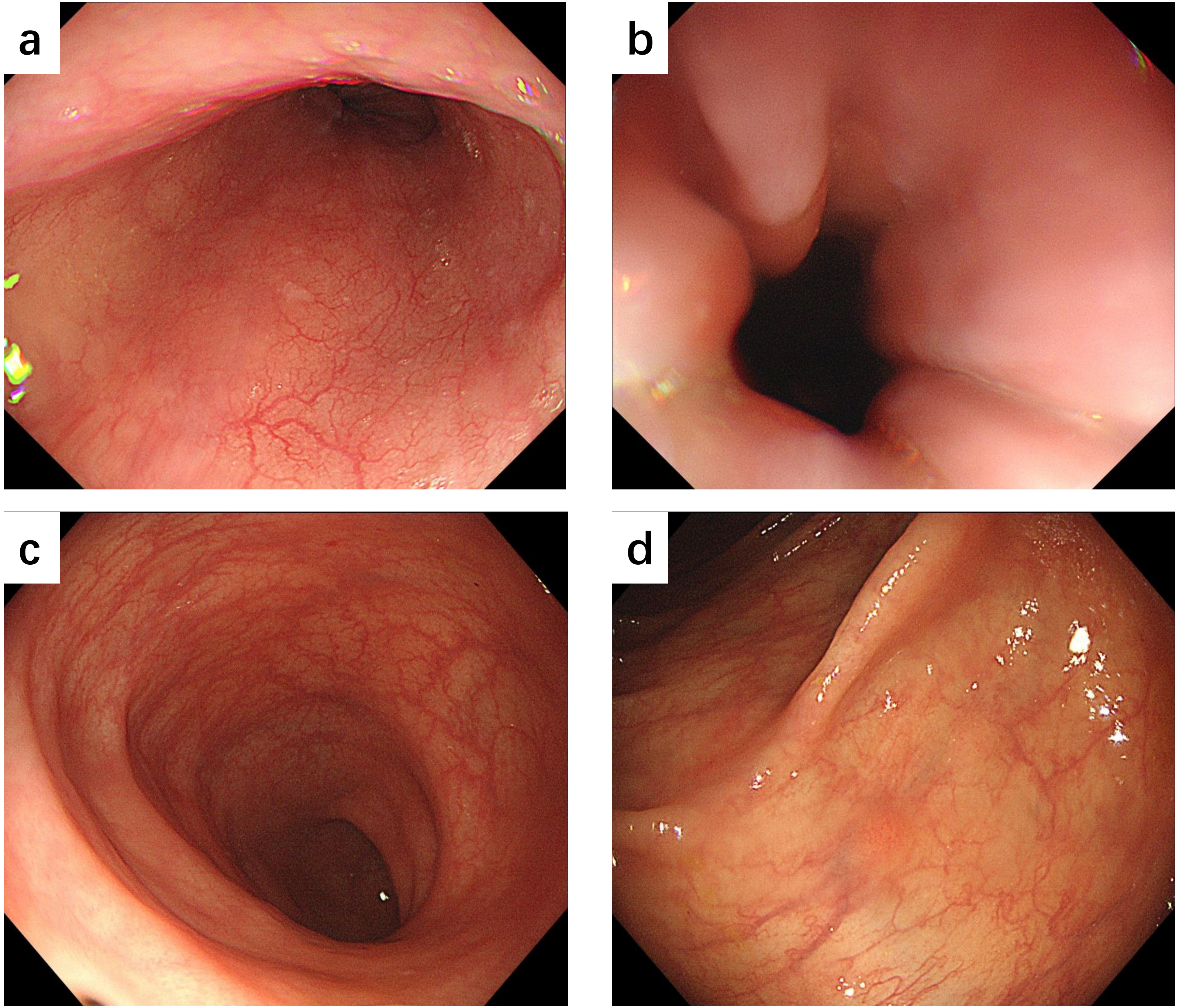

Figure S2. Gastrointestinal endoscopy images after treatment. (a, b) Esophageal mucosa was recovered. (c, d) Colon mucosa was recovered.

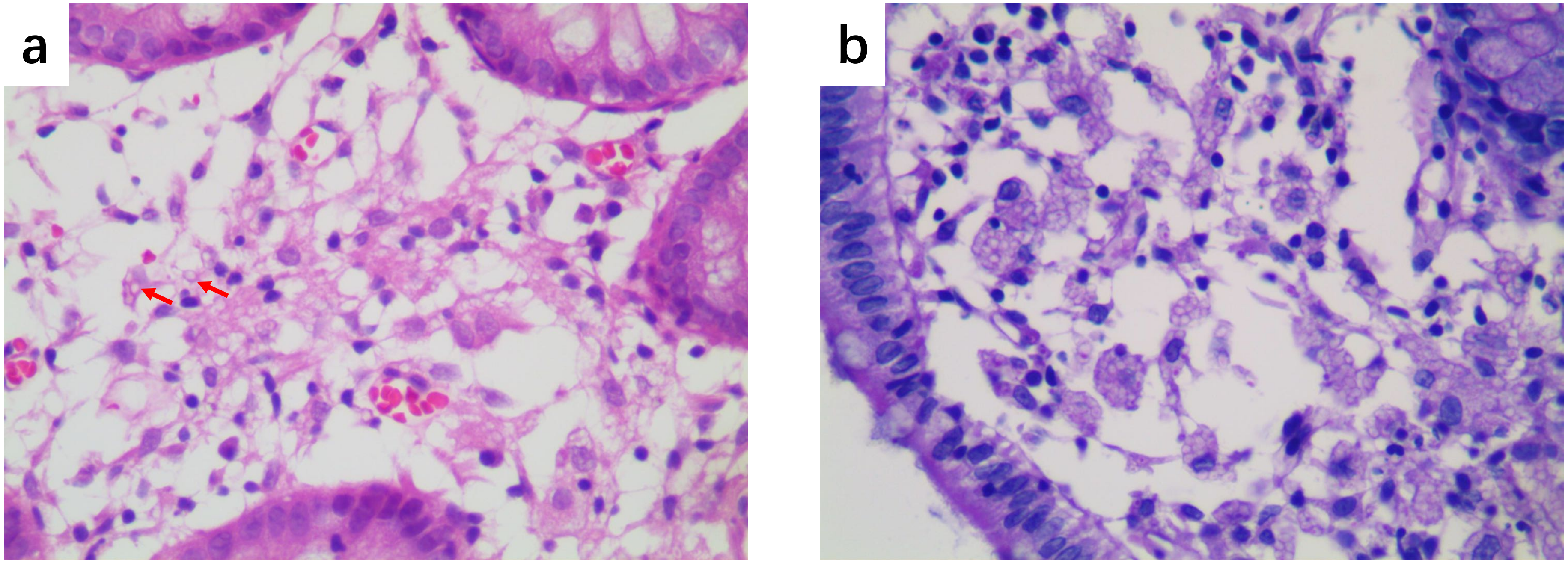

Figure S3. Colonic biopsy images after treatmet. (a) HE stain (red arrows, ×400). (b) PAS stain (negative, ×400).

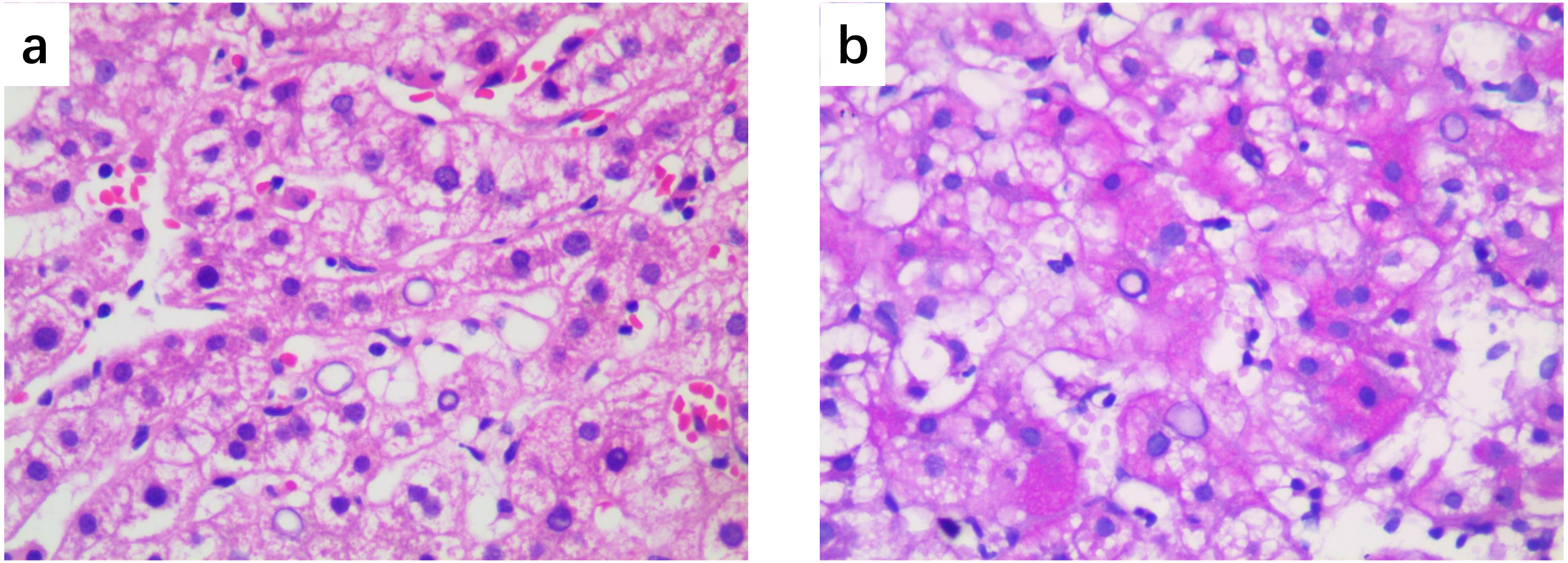

Figure S4. Liver biopsy images. (a) HE stain (×400). (b) PAS stain (negative, ×400).

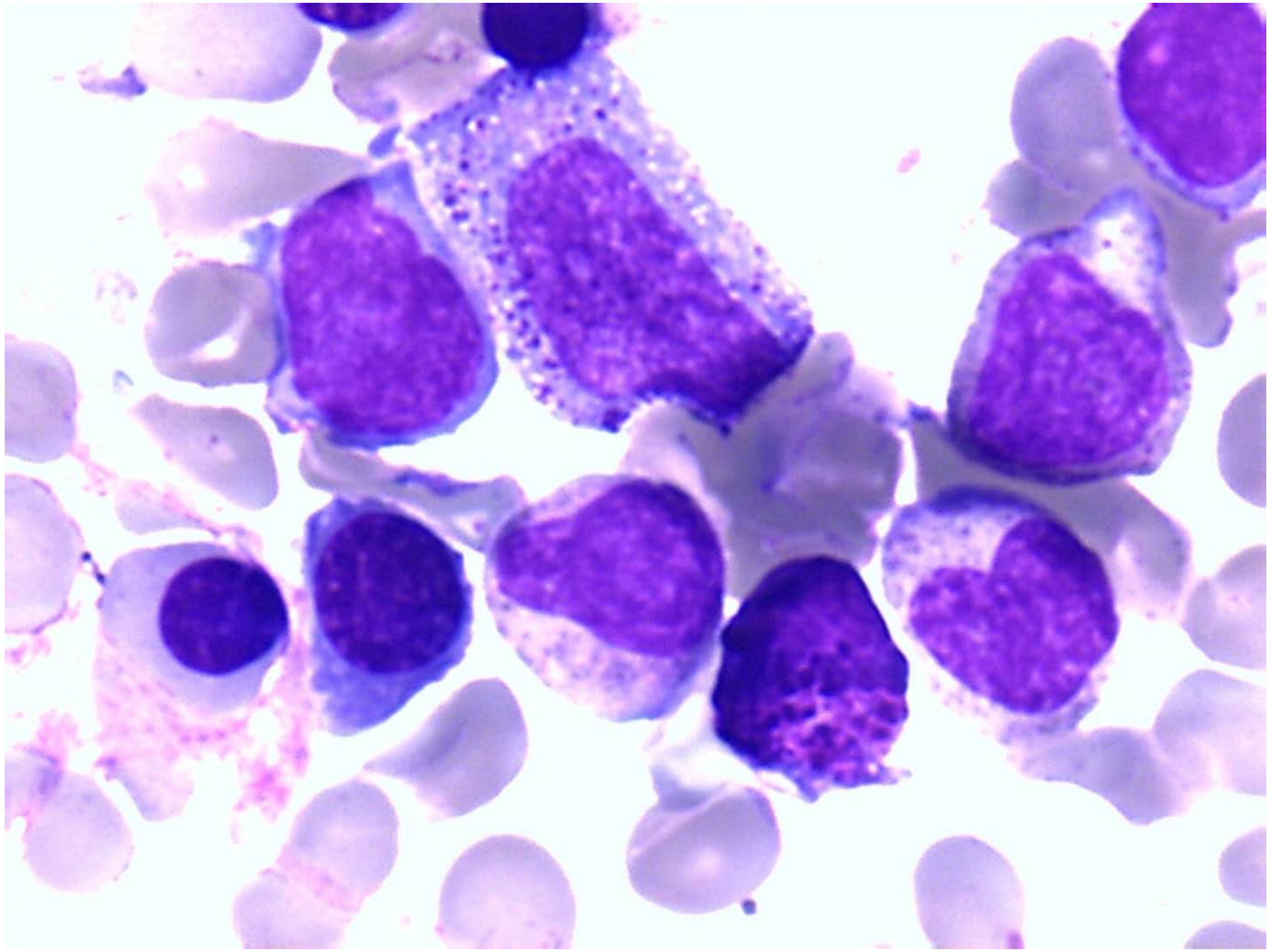

Figure S5. HE stain of bone marrow puncture images after treatment (×1000).
